# Treatment of voluminous and complicated superficial slow-flow vascular malformations with sirolimus (PERFORMUS): protocol for a multicenter phase 2 trial with a randomized observational-phase design

**DOI:** 10.1186/s13063-018-2725-1

**Published:** 2018-06-27

**Authors:** Annabel Maruani, Olivia Boccara, Didier Bessis, Laurent Guibaud, Pierre Vabres, Juliette Mazereeuw-Hautier, Sébastien Barbarot, Christine Chiaverini, Sophie Blaise, Catherine Droitcourt, Stéphanie Mallet, Ludovic Martin, Gérard Lorette, Jean-Baptiste Woillard, Annie-Pierre Jonville-Bera, Jérome Rollin, Yves Gruel, Denis Herbreteau, Dominique Goga, Anne le Touze, Sophie Leducq, Valérie Gissot, Baptiste Morel, Elsa Tavernier, Bruno Giraudeau, Groupe de Recherche de la Societé Française de Dermatologie Pédiatrique

**Affiliations:** 1 University of Tours, University of Nantes, INSERM, SPHERE U1246, Tours, France; 2 Department of Dermatology, Unit of Pédiatric Dermatology, CHRU Tours, 37044 Tours Cedex 9, France; 3 CHRU Tours, Clinical Investigation Center, INSERM 1415, 37000 Tours, France; 4 Department of Dermatology and Reference center for genodermatoses and rare skin diseases (MAGEC), University Hospital Necker-Enfants Malades, 75015 Paris, France; 5 Department of Dermatology, University Hospital Center of Montpellier, 34000 Montpellier, France; 6 University Hospital Center of Lyon, Consultation Multidisciplinaire Lyonnaise des Angiomes, 69229 Lyon Cedex 2, France; 7 Department of Dermatology, University Hospital Center of Dijon, 21000 Dijon, France; 8 Department of Dermatology, University Hospital Center of Toulouse, 31000 Toulouse, France; 9 Department of Dermatology, University Hospital Center of Nantes, 44000 Nantes, France; 10 Department of Dermatology, University Hospital Center of Nice, 06000 Nice, France; 11 Department of Vascular Medicine, University Hospital Center of Grenoble, 38043 Grenoble Cedex 9, France; 12 Department of Dermatology, University Hospital Center of Rennes, 35000 Rennes, France; 13 Department of Dermatology, University Hospital Center of Marseille, 13885 Marseille Cedex 5, France; 14 Department of Dermatology, University Hospital Center of Angers, 49000 Angers, France; 15 Department of Pharmacology and Toxicology, University of Limoges, INSERM UMR 850, CHU Limoges, 87000 Limoges, France; 16 Department of Clinical Pharmacology, Regional Pharmacovigilance Center, CHRU Tours, 37044 Tours Cedex 9, France; 17 Department of Hematology-Hemostasis, University of Tours, UMR-CNRS 7292, CHRU Tours, 37044 Tours Cedex 9, France; 18 Department of Neuroradiology, University of Tours, CHRU Tours, 37000 Tours, France; 19 Department of Maxillo-Facial surgery, University of Tours, CHRU Tours, 37044 Tours Cedex 9, France; 20 Department of Pediatric Surgery, CHRU Tours, 37000 Tours, France; 21 Department of Pediatric Radiology, University of Tours, CHRU Tours, 37000 Tours, France

**Keywords:** Vascular anomalies, Lymphatic malformations, Venous malformations, Children, Sirolimus, Mammalian target of rapamycin inhibitors, Randomized placebo-phase design

## Abstract

**Background:**

Slow-flow superficial vascular malformations (VMs) are rare congenital anomalies that can be responsible for pain and functional impairment. Currently, we have no guidelines for their management, which can involve physical bandages, sclerotherapy, surgery, anti-inflammatory or anti-coagulation drugs or no treatment. The natural history is progressive and worsening. Mammalian target of rapamycin (mTOR) is a serine/threonine kinase that acts as a master switch in cell proliferation, apoptosis, metabolism and angio/lymphangiogenesis. Sirolimus directly inhibits the mTOR pathway, thereby inhibiting cell proliferation and angio/lymphangiogenesis. Case reports and series have reported successful use of sirolimus in children with different types of vascular anomalies, with heterogeneous outcomes.

**Objective:**

The objective of this trial is to evaluate the efficacy and safety of sirolimus in children with complicated superficial slow-flow VMs.

**Methods/design:**

This French multicenter randomized observational-phase, phase 2 trial aims to include 50 pediatric patients 6 to 18 years old who have slow-flow (lymphatic, venous or lymphatico-venous) voluminous complicated superficial VM. Patients will be followed up for 12 months. All patients will start with an observational period (no treatment). Then at a time randomly selected between month 4 and month 8, they will switch to the experimental period (switch time), when they will receive sirolimus until month 12. Each child will undergo MRI 3 times: at baseline, at the switch time, and at month 12. For both periods (observational and treatment), we will calculate the relative change in volume of the VM divided by the study period duration. This relative change weighted by the study period duration will constitute the primary endpoint. VM will be measured by MRI images, which will be centralized and interpreted by the same radiologist who will be blinded to the study period. Hence, each patient will be his/her own control. Secondary outcomes will include assessment of safety and efficacy by viewing standardized digital photographs and according to the physician, the patient or proxy; impact on quality of life; and evolution of biological makers (coagulation factors, vascular endothelial growth factor, tissue factor).

**Discussion:**

The main benefit of the study will be to resolve uncertainty concerning the efficacy of sirolimus in reducing the volume of VMs and limiting related complications and the safety of the drug in children with slow-flow VMs. This trial design is interesting in these rare conditions because all included patients will have the opportunity to receive the drug and the physician can maintain it after the end of the protocol if is found efficient (which would not be the case in a classical cross-over study).

**Trial registration:**

ClinicalTrials.gov Identifier: NCT02509468, first received: 28 July 2015.

EU Clinical Trials Register EudraCT Number: 2015-001096-43.

**Electronic supplementary material:**

The online version of this article (10.1186/s13063-018-2725-1) contains supplementary material, which is available to authorized users.

## Background

### Background and rationale

Vascular anomalies (VAs) include a heterogeneous group of disorders occurring most frequently in newborns and children. Infantile hemangiomas are common (10% of infants), are generally not complicated and are easily managed, but most other VAs are rare (< 2% altogether) and we have no guidelines for management.

The updated classification from the International Society for the Study of Vascular Anomalies (ISSVA), divides VAs into 2 broad categories: vascular tumors and vascular malformations (VMs) [1]. VMs are usually present at birth, although they are not always apparent at this stage; they are distinguished by their hemodynamic flow as high-flow and slow-flow VMs. Slow-flow VMs involve abnormal capillaries vessels (i.e., port-wine stains), venous vessels, lymphatic vessels (including macrocystic, microcystic or mixed lymphatic malformations) or a combination of these. They can be superficial (involving cutaneous and subcutaneous tissues, underlying fasciae and muscles) or may have visceral involvement. They can be limited or diffuse and are sometimes components of genetic syndromes (e.g., Protée, Klippel-Trenaunay, Bean syndrome) [2]. They are always due to defective embryologic vasculogenesis [3]. Slow-flow VMs are diagnosed on physical examination, but a biopsy might be required for confirmation. Diagnosis is always completed with imaging, including duplex ultrasonography for screening, and MRI for definitive diagnosis and diagnosis of extension [4]. Recent molecular findings have allowed for a better understanding of VMs, in particular those for which somatic mutations of genes were identified, especially *PIK3CA*, causing overgrowth tissue associated with the VM [5, 6]. Slow-flow superficial VMs might induce functional impairment because they can be voluminous, painful, associated with underlying overgrowth tissue, or complicated by seepage or continuous cutaneous bleeding. Visceral VMs can be life-threatening when complicated by gastrointestinal bleeding, for instance, or hematologic disturbances (anemia, thrombopenia).

Management of slow-flow VMs requires dedicated multispecialty care. We have no guidelines for treatment, and management may include no intervention (although the natural history of these VMs is progressive worsening), compression with a physical bandage, sclerotherapy, resection (when feasible), anti-inflammatory or anti-coagulation drugs, or interferon or anti-proliferative drugs [7, 8]. Literature reporting medical therapies for slow-flow superficial VMs consists mainly of pediatric case reports and features publication bias, inconsistent use of nomenclature and lack of clinical trials.

Mammalian target of rapamycin (mTOR) inhibitors, usually indicated as immunosuppressive agents for preventing organ rejection [9], have been recently used for VAs, given their anti-proliferative and anti-angio/lymphangiogenic properties, with very promising results [9–12]. They directly inhibit mTOR, a serine-threonine kinase regulated by phosphoinositide-3-kinase (PIK3), which acts as a master switch in numerous cellular processes such as cell growth and proliferation, cellular metabolism, autophagy, and angio/lymphangiogenesis. Inhibitors of mTOR were found efficient in several cases of VAs [12, 13]. A systematic review recorded 84 cases of VAs treated with mTOR inhibitors up to 2014 [14].

Sirolimus (or rapamycin), which has the advantage of oral administration, was the most frequently used mTOR inhibitor and had quick efficacy in children with slow-flow VMs, with overall good tolerance. However, the sirolimus doses, monitoring and outcomes were heterogeneous. An observational study of 61 cases published since then showed more mitigated results [15]. All authors highlighted the need to perform randomized clinical trials to better delineate sirolimus indications in VMs and the modalities of posology and monitoring.

### Objectives

We aimed to perform a clinical trial (*suPERficial slow-flow vascular malFORMations treated with sirolimUS;* PERFORMUS) to assess the efficacy and safety of sirolimus in complicated slow-flow VMs in children.

### Trial design

**Fig. 1:**
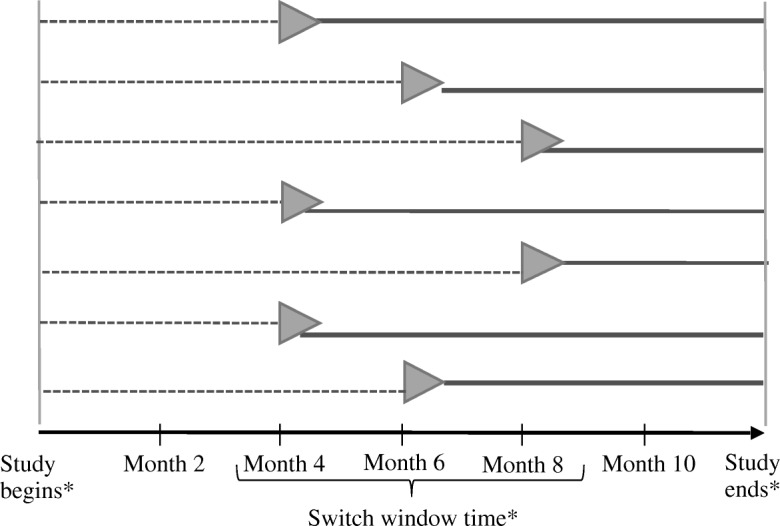
The randomized observational-phase design: data collection for the primary outcome, from MRI

PERFORMUS is a multicentre phase 2 trial with a randomized observational-phase design derived from the design described by Feldman et al. [16]. Patients will be followed up for a total of 12 months. All will start with an observational period (no treatment), then will switch to an experimental period, when they will receive sirolimus. The switch time will be randomized, ranging from month 4 to month 8 (Fig. 1). Such a design was preferred to a two-parallel group design because it allows all children to receive treatment at some time point, thus encouraging physician adherence to the protocol and better patient acceptance. As well, it provides greater power as compared with a two-independent group trial.

## Methods: participants, interventions, and outcomes

### Study setting

The study will be conducted in 12 French tertiary hospital centers involving multidisciplinary consultations (with dermatologists, radiologists and surgeons) dedicated to VAs.

### Eligibility criteria

#### Inclusion criteria

Pediatric patients, age 6 to 18 years, with complicated superficial slow-flow VM extending to underlying tissues (subcutaneous tissue, fasciae, muscles and/or bones) and confirmed by MRI will be eligible. The minimum age of 6 years was chosen to avoid repeated MRI under sedation, as is commonly used for children < 6 years old in most French radiologic centres. Any type of slow-flow VMs, belonging or not to a genetic disorder, might be included, except for pure macrocystic lymphatic malformations, for which sclerotherapy is the usual treatment. Therefore, patients with microcystic lymphatic VMs, mixed micro- and macrocystic VMs, venous malformations or combined lymphatic and venous malformations are eligible if the VM is complicated. Complications might be linked to the volume, pain, functional impairment, bleeding and/or seepage.

#### Exclusion criteria

Patients with life-threatening VM due to complicated visceral involvement will be excluded, as will patients with immunosuppression, a chronic infectious disease or history of cancer in the 2 previous years, contraindications to MRI (prosthesis), pregnancy, and females of childbearing age not using birth control. We will exclude patients with contraindications to sirolimus: allergy to mTOR inhibitor or lidocaine, allergy to peanuts or soybean (only sirolimus pills will be allowed), concomitant treatment that interferes with CYP3A4, concomitant immunosuppressive treatment or previously received an mTOR inhibitor, intolerance to fructose, intolerance or malabsorption to glucose or galactose, metabolic insufficiency in sucrase-isomaltase, metabolic defect in lactase, and liver insufficiency (elevated transaminase level > 2.5 N), anemia (hemoglobulin level < 9 g/dl), leukopenia (white blood cell count < 1000/mm^3^), thrombocytopenia (platelet count < 80,000/mm^3^), or hypercholesterolemia (low-density lipoprotein-cholesterol level ≥ 2 g/l).

### Intervention

Sirolimus will be administered in oral suspension or tablets according to the patient’s age, starting with 0.08 mg/kg/day, max 2 mg/day, twice a day, with dose adjustments based on serum level of sirolimus at day 15 to reach target levels 4 to 12 ng/ml. The maximum dosage will be 6 mg per day. A pharmakocinetics model was developed for sirolimus dose adjustment and was made available for investigators via a secured website (https://pharmaco.chu-limoges.fr/). The model allows for describing and predicting the evolution of drug concentrations based on individual information (measured sirolimus trough level, dose administered and patient weight) and on an a priori set of pharmacokinetics parameters (= the pharmacokinetics population model).

Sirolimus will be supplied to pharmacists at each investigational site and will be in charge of the traceability and storage. The products will be stored at room temperature (maximum 25 °C) for the tablet form and in a cool place (2 to 8 °C) for the oral suspension.

Regarding concomitant treatments, all will be allowed, except for drugs that inhibit CYP3A4 (ketoconazole, voriconazole, itraconazole, telithromycin, clarithromycin) or activate it (rifampcin, rifabutin); and for other concomitant immunosuppressive drugs (to minimize risks of infection).

### Outcomes

#### Primary outcome

The primary outcome is based on the volume of the VM seen on MRI. Patients will undergo 3 MRIs: one at baseline (M0), one at the time of switch from the observational period to the sirolimus period (MS, for “month of switch”) and one at the end of follow-up (M12) (Fig. 1). Relative change in VM volume, divided by the duration period, will define the outcome. Thus, for the observational period, the primary outcome is defined as {(VMS – V0)/V0}/(MS-M0), where V0 and VMS are the volumes assessed at baseline and month S respectively, and (MS-M0) is the duration of the observational period. For the sirolimus period, the outcome is defined in the same way: {(V12 – VMS)/VMS}/(M12-MS), where V12 is the volume assessed at month 12 when the study ends, and (M12-MS) is the duration of the sirolimus period.

### Secondary outcomes

Secondary outcomes are clinical and biological assessments of the efficacy of sirolimus as well as safety.

Clinical efficacy will be assessed by different endpoints (Table 1).

**Table 1:** Schedule of study interventions, outcome data collection and follow-up visits

	Screening	Inclusion	Switch (start sirolimus)	Switch + M1 ± 15 days	Follow-up during sirolimus treatment: every 2 months ±15 days	End of study
	J-30/J0*	J0	Between month 4 and 8**	Between month 5 and 9**	Between month 7 and 11**	Month 12***
Information	x	x				
Inclusion/exclusion criteria	x	x	x	x	x	
Medical history and demographics	x	x				
Consent		x				
Randomization		x				
MRI	x^1^		x			x
Physical examination		x	x	x	x	x
General laboratory tests		x^2^	x^3^	x^4^	x^4^	
Coagulation markers, serum VEGF, tissue factor		x		x	x	x
Serum level of sirolimus adjustments^5^				x	x	
Blood and skin samples for genetic analysis and collection (ancillary study)		x				
Photographs		x	x			x
Self-assessment for patients and proxy by visual analog scale			x	x	x	x
Dermatological quality of life scale		x	x	x	x	x
Adverse events/serious adverse events^6^				x	x	x

*possibly by call phone

**depending on the date of the treatment switch from observational to treatment stage

***sirolimus might be maintained at the discretion of investigators after the protocol is finished

^1^included in routine care

^2^complete blood count, ionogram, creatinine, urea, liver enzymes (gamma-GT, SGOT, SGPT), cholesterol, triglycerides, glucose, infection with HIV and hepatitis B and C, serum β-hCG or urine pregnancy test for women of childbearing age

^3^complete blood count, ionogram, creatinine, urea, liver enzymes, cholesterol, triglycerides, glucose, urinary pregnancy test on women of childbearing age

^4^every month during sirolimus: complete blood count, ionogram, creatinine, urea, liver enzymes, cholesterol, triglycerides, glucose

^5^performed after 15 days of sirolimus and then once a month

^6^adverse events will be recorded from when the informed consent form is signed

*VEGF* vascular endothelial growth factor

Two blinded independent trained readers will qualitatively assess digital photographs. For each patient, readers will be provided photographs at baseline and those at MS and M12 without informative data; they will be asked to identify which photograph was taken at the end of the observational period and which at the end of the sirolimus period. Such a procedure represents a “lady-tasting-tea” procedure [17]. Guidelines will be given to the investigators to perform, with their own camera, a front, a profile and a ¾ photograph of the VM at a 50 cm-distance, with a decimeter placed beside the malformation, the child being standing up or lying. They will be asked to send a copy of the photographs to the Clinical Investigation Center of Tours for further centralized assessment. The photographs will be previously registered with initials of the names, code of the center and inclusion number.

Clinical global improvement will be assessed by an unblinded physician on a 0–10 visual analog scale and will be self-assessed by the patient or proxy (parents) on a 0–10 visual analog scale.

Impact on complications will be self-assessed by the patient or proxy, considering pain and skin complications/symptoms (seepage, bleeding, skin tension, functional impairment) and quality of life by the dermatological quality-of-life scale (DLQI) and DLQI adapted for children (C-DLQI) [18].

Biologic markers of efficacy will include coagulation explorations (platelet count, fibrinogen level, D-dimers, factor V levels), supporting the presence and disappearance of an abnormal intravascular coagulation consumption, and plasma levels of vascular endothelial growth factor (VEGF) and tissue factor. VEGF is a major angiogenic factor that stimulates the proliferation and migration of endothelial cells. Strong expression of VEGF has been found associated with VAs, and a few reports have shown changes in serum levels of VEGF after efficient treatment for VAs and different vascularized cancers [19, 20]. Moreover, Medici et al. demonstrated that sirolimus in vitro inhibits the proliferation of hemangioma endothelial cells by reducing the expression of VEGF [21]. Hence, VEGF could be a useful biologic marker for treatment monitoring. In addition, tissue factor, a 47-kDa glycoprotein that is the physiological trigger of blood coagulation, probably has a key role in modulation of angiogenesis, particularly by regulating VEGF expression [22, 23].

#### Safety

The adverse events (AEs) will be self-declared, through open ended questions which will secondarily be coded. Patients will be questioned about AEs by the investigators, who will particularly check the most frequently AEs reported with sirolimus, i.e. stomatitis, digestive symptoms (nausea, diarrhea), asthenia, cutaneous rash, and infections of mild severity. Lab results are planned before starting sirolimus, then systematically one month after sirolimus is started, then every 2 months until the end of the study (Table 1). Lab results will include complete blood count - the most frequent biological AEs are microcytosis, whose mechanism is not totally elucidated, moderate anemia and decrease in lymphocyte counts -, ionogram, blood lipids, glycemia, and liver and kidney lab tests.

#### Ancillary study

With the organic collection (including blood and skin samples), we will perform genetic analysis of genes involved in vasculogenesis (currently *TIE2* and *PIK3CA*, involved in the mTOR pathway) to allow for a phenotype–genotype association analysis of those VMs [5, 6, 24].

### Participant timeline

Duration of participation will be 12 months for each patient. The time schedule of enrolment and visits is in Table 1.

### Sample size

Because each patient is his/her own control, the randomized observational-phase design is, as a cross-over design, much more powerful than a two-parallel group design. To our knowledge, we have no published data considering both our primary outcome and the target population. We plan to recruit 50 patients. Considering a correlation of 0.5 between the outcomes assessed under the observational and sirolimus period, this sample size will provide the same power as a 200-sample size two-parallel group randomized trial (i.e., 80% power to detect a 0.4 effect size, with a two-sided Type I error of 5%).

### Recruitment

The recruitment of children with these malformations is allowed by the fact that all investigators take part in multidisciplinary consultations dedicated to VAs, including at least dermatologists or other physicians, radiologists, and surgeons. Some of them belong to the French network for research on pediatric dermatology (Groupe de Recherche de la Société Française de Dermatologie Pédiatrique).

## Methods: assignment of interventions

### Allocation

#### Sequence generation and allocation concealment mechanism

Randomization will be handled by a biostatistician not involved in patient recruitment or in their management. We will use a discrete uniform-probability distribution, considering five possible values (i.e., months 4, 5, 6, 7 and 8). Therefore, a randomization sequence with blocks will be generated. No stratification will be considered.

#### Implementation

The allocating sequence will be implemented by means of an electronic Case Report Form (e-CRF): once a patient is included, the physician in charge of this patient will be informed of the treatment switch date.

### Blinding

The trial will be open-label. With the very nature of the design, blinding is artificial in some way. Indeed, everyone knows that the patient begins with an observational period and ends with a period during which they are treated. Moreover, sirolimus requires biologic monitoring to detect AEs and for dose adjustment (by measuring serum level of sirolimus). Blinding would have involved children undergoing numerous useless blood tests and also important logistical constraints because the people managing the sirolimus adjustment would have to be different from care providers. To compensate for the need for an open-label design, the primary outcome data are collected with blinding (i.e., centralized interpretation of MRI images performed by the same radiologist who will be blinded to physical assessment and treatment period).

## Methods: data collection, management, and analysis

### Data collection methods

Table 1 shows data collection according to inclusion and follow-up visits.

### Data management

An e-CRF will be developed by using Ennov Clinical© software. The e-CRF will be managed in agreement with INSERM CIC 1415 Standardized Operating Procedures (SOP). Data from investigating centres will be entered by using a secure web site monitored by clinical research associates, and queries will be edited by data managers, in agreement with an a priori-specified data-management plan. Blinded review will be performed before locking the database. The database will be locked in agreement with INSERM CIC 1415 SOPs and data will be extracted in a SAS format or other, according to statistical requirements. Raw data will be stored in a XML format.

### Statistical methods

Considering the primary outcome, relative changes in volume of VMs will be compared by paired *t* test or Wilcoxon signed-rank test if necessary. For secondary outcomes, physician assessment of efficacy, patient self-assessment and biological relative changes will be analyzed by the same approach (i.e., paired *t* test or Wilcoxon signed-rank test). Considering efficacy on digital photographs, assessed by 2 independent trained readers, the “lady-tasting-tea” procedure will be used to test whether readers performed better than expected in identifying the digital photographs taken after the end of the sirolimus period [17]. Regarding AEs, descriptive statistics (percentages) will be estimated. The ancillary study on genetic samples will be descriptive.

## Methods: monitoring

### Data monitoring

A clinical research technician will be responsible for coordination of the study: will be responsible for the logistics of and monitoring the study; producing reports concerning its state of progress; verifying that the e-CRFs are updated (request for additional information, corrections, etc.); sending blood and skin samples; and transmitting severe AEs to the sponsor. The technician will follow the SOPs.

A data safety monitoring board (DSMB) composed of 3 medical doctors specialized in pharmaco-toxicology, pharmaco-dermatology and pediatric dermatology, is an advisory committee responsible for giving its opinion to the sponsor and the coordinator investigator on the benefit/risk ratio of the trial. The DSMB will be systematically contacted in the following situations: at any time by the sponsor for each case of expected serious adverse reaction or for an suspected unexpected serious adverse drug reaction (SUSAR); before each development safety update report is sent to the French Agency for the Safety of Health Products (ANSM); and if data may change the benefit/risk ratio during the clinical trial.

### Harms

All AEs will be monitored until they are completely resolved. The investigator will immediately notify the sponsor of any serious AE. The sponsor will report all SUSARs to the Eudravigilance (European pharmacovigilance database), French health authorities (ANSM), and the investigators within the regulatory time periods for reporting.

### Auditing

An audit may be performed at any time by sponsor-appointed people who are independent of those responsible for the study. The aim of an audit is to ensure good quality of the study, the validity of results and that the law and regulations in force are well observed. The investigators agree to comply with the requirements of the sponsor and the relevant authority for an audit or inspection of the study. The audit can apply at all stages of the study, from development of the protocol to publication of results.

## Ethics and dissemination

### Research ethics approval

The sponsor and the investigators undertake to conduct this study in compliance with French law no. 2004–806 of August 9, 2004 and following Good Clinical Practice and the Helsinki Declaration (Ethical Principles for Medical Research involving Human Subjects, Tokyo 2004). The study will be conducted in accordance with this protocol. With the exclusion of emergency situations requiring specific therapeutic actions, the investigators will observe the protocol in all respects, particularly in obtaining consent and the notification and follow-up of serious AEs.

The protocol was approved by the institutional review board of the University Hospital Centre of Tours (#2015-R18) and received authorization from ANSM.

### Protocol amendments

Important protocol modifications will be submitted as well for approval to the Institutional review board of the University hospital of Tours, and will be communicated to coinvestigators.

### Consent and assent

Both parents will give their informed signed consent after their child has consented (if able) and they have been orally informed of the study and have received a written information form (Additional file 1). These potential participants are informed that they are free to withdraw from the study at any moment.

### Confidentiality

During this biomedical research study or when it is over, the information collected on the people taking part in it and forwarded to the sponsor by the investigators (or any other specialized staff member involved) will be made anonymous. Under no circumstances will the uncoded names or addresses of the people concerned appear in any data.

### Access to data

The sponsor is responsible for obtaining agreement from all the parties involved in the study in order to guarantee direct access, in all the sites where the study is being conducted, to source data, source documents and reports, to control their quality and to audit them.

The investigators will make available to people with a right of access to these documents, according to the legislative and regulatory provisions in force (articles L.1121–3 and R.5121–13 of the French Public Health Act), the documents and individual data strictly necessary for monitoring, carrying out quality control and auditing the biomedical research.

### Dissemination policy

INSERM CIC 1415 Tours will analyze the data provided by the study centres. Results will be displayed in a written report that will be submitted to the sponsor. At the end of the analysis, results will be published in ClinicalTrials.gov. The international rules for writing and publication (Vancouver Agreement, February 2006) will be followed.

In accordance with law no. 2002–303 of March 4, 2002, patients will be informed, at their request, about the overall results of the study [25].

### Spirit

This protocol has been written in accordance with the Standard Protocol Items: Recommendations for Interventional Trials (SPIRIT) guidelines. The SPIRIT checklist is in Additional file 2. The SPIRIT figure is in Additional file 3.

## Discussion

A randomized trial on sirolimus in VMs was needed, but we had to overcome several difficulties to design such a study: the population is too rare to allow for a parallel-group trial; we have no gold standard for comparing sirolimus; and a placebo group allowing for blinding is not acceptable in this pediatric population because biological samples are required for sirolimus monitoring.

The “randomized observational-phase design” is adapted from the “randomized-placebo phase design” published by Feldman, developed to suit rare conditions for which a placebo group is difficult to obtain. Hence, the design is particularly adapted to rare pediatric conditions. In this design, the subject is his/her own control, as in cross-over trials. However, a cross-over design would have involved the group of patients who receive sirolimus in the first phase to stop it in the second phase, even if it was beneficial. Moreover, cross-over design supposes that after the end of each treatment period, the disease returns to the initial state, which is perhaps not the case here, in that sirolimus might offer lasting improvement.

This randomized observational-phase trial also allows for maintaining sirolimus after the 12-month period of the trial if it is efficient. One of its potential drawbacks is premature termination of treatment. In case this happens, a final assessment (an MRI in our example) will have to be performed.

Because patients and care providers are not blinded to the treatment during that period, the primary outcome was chosen to be as objective as possible (i.e., changes in the volume of the VM seen on MRI). However, in practice, patients complain more about symptoms and complications linked to the VM, such as pain, oozing, bleeding, or functional impairment, than about the volume of the VM. Therefore, assessment of clinical efficacy and self-assessment on symptoms and complications are secondary outcomes of high importance. Safety data will be a focus in this pediatric phase 2 trial, because decisions for treatment in other children with VMs will be based on the benefit/risk ratio.

Hence, the main benefit of the study will be to resolve uncertainty concerning the efficacy and safety of sirolimus in children with slow-flow VMs, a subgroup of VAs that includes heterogeneous entities. If we demonstrate a benefit of sirolimus, the remaining question will be how long treatment has to be maintained.

### Trial status

The protocol is currently recruiting in 12 French centres. The first patient was included on December 30, 2016, and recruitment is anticipated to end in March 2018, with follow-up completed in March 2019.

## Additional files

Additional file 1: Informed consent materials. (PDF 122 kb)

Additional file 2: SPIRIT checklist. (PDF 109 kb)

Additional file 3: SPIRIT figure. (DOC 50 kb)

## Abbreviations

AEs: Adverse Events
ANSM: Agence Nationale de Sécurité du Médicament et des produits de santé
CYP2A4: Cytochrome P450, family 2, subfamily a
DSMB: Data Safety Monitoring Board
e-CRF: Electronic Case Report Form
MRI: Magnetic Resonance Imaging
mTOR: Mammalian Target Of Rapamycin
SAEs: Severe Adverse Events
SOP: Standardized Operating Procedures
SUSAR: Suspected Unexpected Serious Adverse Reaction
VA: Vascular Anomaly
VEGF: Vascular Endothelial Growth Factor
VM: Vascular Malformation
