# Supplementary material for: Treatment of voluminous and complicated superficial slow-flow vascular malformations with sirolimus (PERFORMUS): protocol for a multicenter phase 2 trial with a randomized observational-phase design
Source: Trials. 2018 Jun 27;19:340. doi: 10.1186/s13063-018-2725-1 (PMC6020321; doi:10.1186/s13063-018-2725-1)
Supplement: Supplementary file 1 — Informed consent materials. (PDF 122 kb) [file 13063_2018_2725_MOESM1_ESM.pdf]

CONSENT FORM - FORMULAIRE DE CONSENTEMENT DE PARTICIPATION À UNE RECHERCHE BIOMÉDICALE : *ÉTUDE PERFORMUS*

Le Dr/Pr…………………………………….............. (Service de ………………………………………., tél ………………..…………), médecin investigateur, m'a proposé de participer à une étude clinique organisée :

► **par le CHRU de TOURS, 2, bd Tonnellé 37044 Tours cedex 9**

► **intitulée « Traitement des malformations vasculaires superficielles complexes à bas debit par sirolimus : essai thérapeutique de phase II chez l'enfant ».**

Après avoir reçu oralement et par écrit toutes les informations nécessaires pour comprendre l'intérêt de cette étude, de ses contraintes et de ses risques éventuels, ainsi que ce qu'il est demandé de faire dans le cadre de la participation, et ayant pu poser toutes les questions au médecin investigateur ; sachant que :

- Je suis libre de refuser ou de décider à tout moment de ne plus participer : sachant que je peux à tout moment obtenir des informations complémentaires auprès du médecin investigateur.
- Le médecin a le droit d'interrompre la participation à l'étude s'il pense que cela est bénéfique pour mon enfant.
- Cette étude est menée en conformité avec le Code de la Santé Publique, titre II du livre premier relatif aux recherches biomédicales.
- Conformément à la loi, le CHRU de Tours, promoteur de cette étude a souscrit une assurance de responsabilité auprès de la compagnie SHAM (Société Hospitalière d'Assurances Mutuelles – 18 rue E Rochet – 69372 Lyon cedex 08) afin de couvrir tout préjudice corporel ou toute incapacité que pourrait entraîner cette étude.

**A compléter par les parents /responsables légaux**<br>Je soussignée NOM…………………………………………<br>Prénom………………………………………<br>(mère de l'enfant)<br>Et/ou<br>Je soussigné Nom<br>…………………………...Prénom………………………………………<br>(Père de l'enfant)<br>déclarons(e) être en d'accord avec les dispositions de la présente attestation de consentement et déclarons(e) donner librement notre (mon) consentement éclairé pour que notre enfant participe à cette recherche biomédicale.	**Date :**<br><br>**Signature :**
**A compléter par l'enfant EN AGE DE DONNER SON ACCORD**<br>Moi, (Prénom)……………………….. accepte de participer à cette étude	**Date :**<br><br>**Signature :**
**A compléter par le médecin-investigateur**<br>Je soussigné(e) Pr/Dr NOM………………………….<br>PRENOM…………………………<br>Déclare avoir personnellement recuilli le consentement de participation des (du) parent(s) de cet(te) enfant, à cette recherche biomédicale.	**Date :**<br><br>**Signature :**

- Cet accord ne décharge en rien les organisateurs de l'étude de leur responsabilité à mon égard.

- Conformément aux dispositions de la loi Informatique et Libertés précitée, je dispose d'un droit d'accès et de rectification des données personnelles me concernant détenues par le responsable du fichier de traitement informatisé mis en place dans le cadre de cette étude. Je dispose aussi du droit de m'opposer à la transmission des données couvertes par le secret professionnel. Ces droits s'exercent auprès du médecin qui me suit dans le cadre de cette recherche et qui connaît mon identité. Conformément à l'article L. 1111-7 du code de la santé publique, je pourrai exercer mon droit de regard et de rectification sur les données me concernant par l'intermédiaire du médecin de mon choix.

- J'accepte le traitement informatisé des données nominatives qui me concernent en conformité avec les dispositions de la loi 78-17 « Informatique, fichiers et Libertés » du 6 janvier 1978 modifiée le 6 août 2004. Mon identité restera confidentielle et les données me concernant seront codées. Elles ne pourront être consultées que par les organisateurs de l'étude ou leurs représentants ainsi que par des membres des Autorités de Santé. Conformément à l'article L. 1111-7 du code de la santé publique, je pourrai exercer mon droit de regard et de rectification sur les données me concernant par l'intermédiaire du médecin de mon choix.

	**Investigateur**<br>(ou médecin qui le représente)	Représentant légal 1<br>(parent ou tuteur)	Représentant légal 2<br>(parent ou tuteur)	Mineur participant à la recherche
**Date**	Fait à Tours, le …...................	à Tours, le ….......	à Tours, le …..............	à Tours, le …...........
**Signature**				

► **Paragraphe relatif aux analyses génétiques et à la conservation de sang pour une collection biologique**

Dans le cadre de l'étude PERFORMUS, il est proposé une analyse génétique sur un prélèvement sanguin et cutané, ainsi qu'un prélèvement qui sera conservé pour une collection biologique, afin de réaliser des analyses génétiques ultérieures, dans le cadre de la pathologie de votre enfant. Cette collection sera réalisée conformément à la réglementation et déclarée auprès des instances.
Vous pouvez refuser que votre enfant participe à l'analyse génétique et/ou à cette collection et demander la destruction de ses échantillons à tout moment.
Pour ces recherches votre accord écrit est nécessaire. Vous pouvez l'exprimer en signant le présent document.
Votre consentement est révocable à tout moment. Après avoir lu cette note d'information, n'hésitez pas à poser à votre médecin toutes les questions que vous désirez.

Nous soussignés, Mme... (*nom, prénom) et* M ....

...........(*nom, prénom),* parents de .... *(nom, prénom de l'enfant)* autorisons le

Pr/Dr……………………..

à faire effectuer les prélèvements de sang et de peau qui serviront à un examen des caractéristiques génétiques de mon enfant (cocher en cas d'accord)

à faire le prélèvement de sang sur mon enfant pour le conserver dans une collection biologique, à des fins scientifiques ultérieures (cocher en cas d'accord)

Fait à …………………………

le |__|__| |__|__| |__|__|__|__|

Signature des deux titulaires de l’autorité parentale :

Signature du médecin
