# Supplementary material for: Treatment of voluminous and complicated superficial slow-flow vascular malformations with sirolimus (PERFORMUS): protocol for a multicenter phase 2 trial with a randomized observational-phase design
Source: Trials. 2018 Jun 27;19:340. doi: 10.1186/s13063-018-2725-1 (PMC6020321; doi:10.1186/s13063-018-2725-1)
Supplement: Supplementary file 3 — SPIRIT figure. (DOC 50 kb) [file 13063_2018_2725_MOESM3_ESM.doc]

Additional file 3: SPIRIT Figure

|  | **STUDY PERIOD** | | | | | |
| --- | --- | --- | --- | --- | --- | --- |
|  | **Screening** | **Inclusion** | **Switch (start sirolimus)** | **Switch + M1 +/- 15 days** | **Follow-up during sirolimus treatment: every 2 months +/- 15 days** | **Close-out** |
| **TIMEPOINT**** | ***J-30/J0**** | **J0** | ***Between month 4 and 8***** | ***Between month 5 and 9***** | ***Between month 7 and 11***** | ***Month 12****** |
| **ENROLMENT:** |  |  |  |  |  |  |
| **Eligibility screen** | X |  |  |  |  |  |
| **Informed consent** |  | X |  |  |  |  |
| **Randomization** |  | X |  |  |  |  |
| **ASSESSMENTS:** |  |  |  |  |  |  |
| ***Primary outcome*** |  | | | | | |
| **MRI** | X^1^ |  | X |  |  | X |
| ***Secondary outcomes*** |  | | | | | |
| **General laboratory tests** |  | X^2^ | X^3^ | X^4^ | X^4^ |  |
| **Coagulation markers, serum VEGF, tissue factor** |  | X |  | X | X | X |
| **Blood and skin samples for genetic analysis and collection (ancillary study)** |  | X |  |  |  |  |
| **Photographs** |  | X | X |  |  | X |
| **Self-assessment for patients and proxy by visual analog scale** |  |  | X | X | X | X |
| **Dermatological quality of scale** |  | X | X | X | X | X |
| **Adverse events/serious adverse events^5^** |  |  |  | X | X | X |

**depending on the date of the treatment switch from observational to treatment stage

***sirolimus might be maintained at the discretion of investigators after the protocol is finished

^1^included in routine care

^2^complete blood count, ionogram, creatinine, urea, liver enzymes (gamma-GT, SGOT, SGPT), cholesterol, triglycerides, glucose, infection with HIV and hepatitis B and C, serum β-hCG or urine pregnancy test for women of childbearing age

^3^complete blood count, ionogram, creatinine, urea, liver enzymes, cholesterol, triglycerides, glucose, urinary pregnancy test on women of childbearing age

^4^every month during sirolimus: complete blood count, ionogram, creatinine, urea, liver enzymes, cholesterol, triglycerides, glucose

^5^adverse events will be recorded from when the informed consent form is signed

*VEGF: vascular endothelial growth factor*
